# Sleep-Disordered Breathing Symptoms and Central Adiposity in Children with Adenotonsillar Hypertrophy

**DOI:** 10.3390/jcm15145347

**Published:** 2026-07-08

**Authors:** Wioleta Umławska, Katarzyna Pawłowska-Seredyńska, Monika Krzyżanowska, Katarzyna Resler, Marcin Frączek, Monika Morawska-Kochman

**Affiliations:** 1 Department of Human Biology, University of Wroclaw, 50-137 Wroclaw, Poland; wioleta.umlawska@uwr.edu.pl (W.U.); katarzyna.pawlowska@uwr.edu.pl (K.P.-S.); monika.krzyzanowska@uwr.edu.pl (M.K.); 2 Faculty of Dentistry, Wroclaw Medical University, 50-367 Wroclaw, Poland; katarzyna.resler@umw.edu.pl; 3 Faculty of Medicine, Wroclaw Medical University, 50-367 Wroclaw, Poland; marcin.fraczek@umw.edu.pl

**Keywords:** adenotonsillar hypertrophy, sleep-disordered breathing, children, adiposity, body fat distribution, paediatric obesity

## Abstract

**Background/Objectives:** Children with adenotonsillar hypertrophy (ATH) and sleep-disordered breathing (SDB) may have altered body composition. The roles of anatomical upper airway obstruction and functional respiratory impairment in the risk of body composition remain poorly understood. This study assessed nutritional status in children. It also examined how nasopharyngeal obstruction (NP) due to ATH and SDB symptoms affects adiposity and fat distribution. **Methods:** This cross-sectional study included 106 children (57 boys, 49 girls; mean age 6.1 ± 2.0 years) who were scheduled for surgical treatment. Flexible nasopharyngolaryngoscopy was used to measure the size of the adenoid and palatine tonsils and combined grades of adenoid and tonsillar hypertrophy were used to determine NP obstruction. Parents filled out a questionnaire to assess SDB symptoms. Anthropometric measures (height, weight, BMI, skinfold thicknesses, body circumferences) were expressed as age- and sex-standardised scores. General linear models (GLMs) were used to examine associations between disease factors and body composition. **Results:** About 21% of participants were classified as overweight or obese. In contrast, 10% were undernourished. Severe NP obstruction was present in 57% of children. SDB symptoms appeared in 28% of children. Despite normal linear growth, children with ATH showed greater adiposity than reference values. In univariate analyses, severe NP obstruction was associated with higher body weight, BMI, triceps and subscapular skinfold thicknesses, waist circumference, and waist-to-height ratio (WHtR). However, after adjustment for BMI, these associations were no longer significant. In contrast, SDB symptoms were independently associated with increased subcutaneous adiposity, as indicated by greater subscapular and abdominal skinfold thicknesses, greater mid-upper arm fat area, and higher WHtR. The pattern showed a more central distribution of fat. **Conclusion:** Children with ATH and SDB symptoms reported by a parent had increased subcutaneous and central adiposity, independent of BMI or the anatomical severity of nasopharyngeal obstruction. Longitudinal studies incorporating objective assessment of SDB are warranted to further clarify the mechanisms underlying these associations.

## 1. Introduction

Adenoid and tonsillar hypertrophy is one of the most common causes of upper airway obstruction in children and is seen with a wide range of clinical symptoms, including nasal obstruction, recurrent infections, snoring, otitis media and growth failure [1,2,3,4,5]. In many cases, adenotonsillar hypertrophy (ATH) leads to sleep-disordered breathing (SDB), ranging from habitual snoring to obstructive sleep apnea syndrome (OSAS) [6]. SDB in childhood has been associated with adverse neurocognitive, behavioural, cardiovascular and metabolic outcomes. Early identification and appropriate management of children is therefore important [7,8,9,10].

The evaluation of upper airway obstruction in children is mainly based on assessment of adenoid and tonsillar hypertrophy and clinical symptoms such as snoring, mouth breathing, and witnessed sleep apnea episodes [11]. However, the relationship between anatomical enlargement and the severity of functional impairment remains complex and is not always directly proportional [12,13]. In normal-weight children, a moderate association has been reported between adenotonsillar size and the apnea–hypopnea index (AHI), whereas upper airway collapsibility assessed by the Mallampati score seems to be a stronger predictor of OSAS in obese children, suggesting differing underlying pathophysiological mechanisms [14,15]. In particular, central adiposity is of major importance for the genesis and advancement of OSAS [16]. Adenotonsillectomy is usually associated with improved respiratory outcomes, particularly in obese patients. However, residual disease is common and post-operative follow-up, including weight management, is important [17].

Anthropometric assessment in the paediatric setting is often performed using body mass index (BMI); however, BMI does not accurately reflect body fat composition or distribution [18]. Thus, other parameters, such as skinfold thickness and circumferences of the neck, arms, waist, and hips, and indices such as waist-to-hip ratio (WHR) and waist-to-height ratio (WHtR), can better assess adiposity. Such strategies may be particularly useful for children with sleep-related breathing disorders [19].

Previous studies suggest that children with ATH and SDB may have altered body composition. However, the relative contribution of anatomical upper airway obstruction and functional respiratory impairment to nutritional status and related health complications is poorly understood [20,21,22]. Therefore, the present study aimed to: (1) assess nutritional status and body fat distribution in children with ATH who were qualified for surgical treatment, and (2) evaluate the impact of nasopharyngeal obstruction due to ATH and SDB on the body adiposity and fat distribution in this group.

## 2. Material and Methods

### 2.1. Subjects/Patients

The study was conducted at a tertiary otolaryngology–head and neck surgery centre in Poland between July 2013 and 2014. The study protocol was approved by the Bioethics Committee of Wroclaw Medical University, Poland (Approval No. KB 344/2013, approved on 27 June 2013), before patient enrolment commenced, and was conducted in accordance with the principles of the Declaration of Helsinki. Written informed consent was obtained from the parents or legal guardians of all participating children. Clinical and anthropometric data were collected on 139 children referred to the paediatric otolaryngology department for adenoidectomy or adenotonsillectomy. All children were considered surgical candidates according to international guidelines, including those of the American Academy of Otolaryngology–Head and Neck Surgery and UK clinical guidelines. Inclusion criteria were: (1) recurrent or chronic tonsillitis with a frequency and severity of symptoms consistent with established criteria, and/or (2) obstructive sleep-disordered breathing associated with adenotonsillar hypertrophy and related symptoms (i.e., nasal obstruction, snoring, apnea, recurrent upper respiratory tract infections, hearing impairment or recurrent otitis media) [23].

Thirty-three of these children were excluded due to missing essential data or the presence of other growth-related diseases (e.g., genetic diseases, endocrine disorders, kidney failure) or SDB-related conditions (e.g., congenital craniofacial abnormalities). Finally, 106 children (57 boys, 49 girls; mean age 6.1 ± 2.0 years) were enrolled for the analysis.

### 2.2. Clinical Assessment

The size of the adenoid and palatine tonsils was evaluated endoscopically using flexible nasopharyngolaryngoscopy. The degree of adenoid hypertrophy was assessed on a 3-point scale: <50% obstruction was grade 1, 50–75% was grade 2, and >75% was grade 3 [24]. For analysis, grades 1 and 2 were grouped as low-grade adenoid hypertrophy, while grade 3 was classified as severe adenoid hypertrophy. The degree of hypertrophy of the tonsils was assessed using the 4-point Brodsky grading scale; grades 0–2 were categorised as small tonsils, while grades 3 and 4 were classified as large tonsils [25]. Because the grades of adenoid and tonsillar hypertrophy, considered separately, do not fully explain the degree of upper airway obstruction, we summarised the grades of adenoid and tonsillar hypertrophy. Patients with the sum of adenoid and tonsillar hypertrophy grades < 4 were considered a group with moderate nasopharyngeal obstruction, while children with the sum of adenoid and tonsillar hypertrophy grades ≥ 4 were considered a group with severe NP obstruction.

The history of previous adenoidectomy and/or tonsillotomy procedures, age at onset of first symptoms (in years), and disease duration (in years) were obtained from clinical records and parental medical history. Parental anamnesis was used to assess the presence of SDB symptoms in the study participants. Parents of the participating children completed a custom-designed questionnaire assessing the presence of sleep disturbances. The questionnaire included items on nighttime symptoms such as snoring, observed apneic episodes (breathing pauses during sleep), restless sleep, and morning headaches.

Responses were recorded on a four-point scale: ‘never’, ‘rarely’, ‘frequently’, and ‘very frequently’. In this study, frequent or very frequent occurrence of all listed symptoms was interpreted as indicative of obstructive sleep-disordered breathing and considered a potential marker of increased risk for SDB in the study population.

### 2.3. Somatic Measurements, Anthropometry

All anthropometric measurements were performed by trained research staff to reduce the inter-measurer variability. Measurements were taken after an overnight fast and the children were lightly dressed. Body height was measured to an accuracy of 0.1 cm with Martin’s anthropometer (Alumet Inc., Warsaw, Poland). Body weight was measured to an accuracy of 0.1 kg. Three skinfold thicknesses (SFTs)—tricipital SFT, subscapular SFT, and abdominal SFT—were measured up to 0.2 mm with a H.oltain calliper (Baty International Ltd., Burgess Hill, UK). Mid-upper arm circumference (MUAC) was measured midway between the acromion and ulnar processus up to 0.1 cm, using anthropometric tape (Seca gmbh&co, Hamburg, Germany)). Waist circumference (WC) was measured at the minimum circumference between the iliac crest and the rib cage with an anthropometric tape at the end of normal expiration to the nearest 0.1 cm.

Based on the somatic measurements performed, the following nutritional status indicators were calculated: body mass index (BMI, in kg/m^2^), the sum of three SFTs (in mm), and three mid-arm cross-sectional areas (in mm^2^): mid-upper arm area (MUAA), mid-upper arm muscle area (MUAMA), and mid-upper arm fat area (MUAFA). The waist-to-height ratio (WHtR) was used to assess adipose tissue distribution.

All anthropometric parameters and indices (except WHtR) were presented as age- and sex-specific standard deviation scores (SDSs) according to the growth references for Polish children [26,27]. The WHtR values were not standardised due to the lack of appropriate reference norms for Polish children.

The prevalence of obesity, normal body weight, and underweight among the study participants was estimated using BMI cutoff points proposed by the Obesity Task Force and by Cole et al. [28,29]. The cutoff values for moderate and severe underweight are 18.5 kg/m^2^ and 17 kg/m^2^, respectively, in adults, while those for overweight and obesity are 25 kg/m^2^ and 30 kg/m^2^, respectively.

### 2.4. Statistical Analysis

Analyses were performed using STATISTICA 13.3 software (StatSoft Ltd., Krakow, Poland). Anthropometric measurements and indices, age, and age at onset of the first disease symptoms were presented as means and standard deviations. Disease duration, adenoid and tonsil sizes, NP obstruction, presence of SDB symptoms, and previous tonsillotomy or tonsillectomy were treated as dichotomous qualitative variables. The quantitative data were tested for normality using the Shapiro–Wilk test. Differences in growth and nutritional status between the study group and reference population were tested with Student’s *t*-test for a single sample. Differences in quantitative variables between two independent groups were assessed using the independent-sample Student’s *t*-test or the Mann–Whitney U test. Pearson’s correlation coefficient was used to analyse associations between continuous variables, while categorical associations were assessed with the Chi-square test with Yates’ correction. A general linear model (GLM) was applied to assess the effects of selected disease-related factors on nutritional status and the distribution of subcutaneous adipose tissue. The goodness-of-fit of the multiple regression model was assessed using the adjusted coefficient of determination (adjusted R^2^), which accounts for the number of predictors. Differences were considered statistically significant at *p* < 0.05.

## 3. Results

### 3.1. Characteristics of Subjects

A total of 106 children (57 boys and 49 girls), aged 2.5–12.3 years (mean age 6.1 ± 2.0 years), were included in the study. The mean age at symptom onset associated with adenoid and adenotonsillar hypertrophy was 3.4 ± 2.1 years. In most patients (76%), the disease duration was less than 3 years. Severe adenoidal hypertrophy was observed in 42% of children, while large tonsils were present in 33% of the participants. After a combination of adenoid and tonsillar hypertrophy grades, we observed severe NP obstruction in about 57% of the study group.

A history of adenoidectomy or tonsillectomy was reported in 13% of children. SDB symptoms were present in 28% of participants. No significant differences were found between boys and girls in disease characteristics or the clinical course of the disease (Table 1).

### 3.2. Growth and Body Composition Characteristics

**Figure 1:**
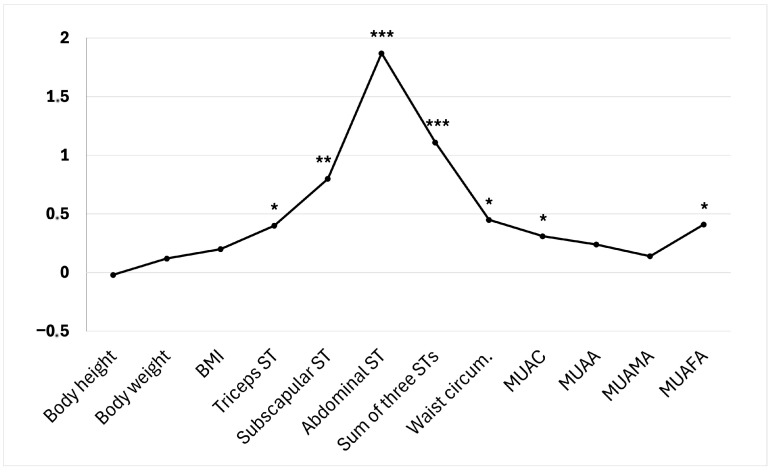
Mean values (+/− SD) of sex- and age-adjusted anthropometric indices (z-scores) of children and statistical significances of differences in anthropometry between patients and reference population according to one-sample *t*-test (* *p* < 0.05, ** *p* < 0.01, *** *p* < 0.001). MUAA—mid-upper arm area; MUAC, mid-upper arm circumference; MUAFA—mid-upper fat area; MUAMA—mid-upper arm muscle area; MUAFA—mid-upper arm fat area, ST, skinfold thickness.

Based on BMI assessments, slightly less than 70% of the examined children were classified as having a normal body weight. Undernutrition was identified in 10% of participants, while overweight or obesity was observed in 21% of the study population. Children with ATH did not differ from healthy peers in linear growth; however, they had a higher body fat percentage. All measured skinfold thicknesses, the sum of skinfolds, mid-upper arm circumference (MUAC), upper arm fat area, and waist circumference were significantly greater in the study group than in the reference group (Figure 1).

No significant differences were observed between boys and girls in somatic indices, except for standardised mean body height, which was significantly higher in boys.

### 3.3. Relationship Between Disease-Related Factors and Body Composition

First, correlations between patients’ somatic indices and clinical variables were evaluated. As no significant differences in most of the somatic indices were found between girls and boys, all analyses were performed on the combined sample (Table 2).

Two clinical variables were significantly associated with the children’s somatic parameters: the degree of NP obstruction and the presence of SDB symptoms. Children with severe NP obstruction had significantly higher mean adjusted body weight, BMI, waist circumference, triceps SFT and sum of SFTs than those with mild or moderate NP obstruction. Moreover, SDB-symptomatic subjects showed increased subcutaneous adiposity, with a clear central fat deposition.

Greater adenoidal hypertrophy was more frequently observed in children with a shorter than a longer disease history (47% vs. 24%; *p* < 0.000).

No significant relationship was found between the severity of NP obstruction and SDB symptoms (Table 2).

### 3.4. Multivariate General Linear Model (GLM) Analysis

Table 3 presents the results of the general linear model (GLM). The model included only predictors that were statistically significant in univariate analyses evaluating nutritional status and adipose tissue distribution patterns. These predictors included the degree of NP obstruction and the presence of SDB symptoms. BMI was included as a covariate to control for the effect of overall body weight on body composition. No significant associations were found between the degree of NP obstruction and anthropometric variables.

On the other hand, symptoms of SDB in children were positively associated with subscapular and abdominal skinfold thickness and the sum of skinfolds thicknesses. Also, significant positive associations were found with mid-upper arm fat area (MUAFA) and waist-to-height ratio (WHtR). These findings indicate that children with ATH and those with SDB symptoms exhibit increased subcutaneous adiposity, particularly in the trunk region.

## 4. Discussion

In this study, we examined the association between disease severity and nutritional status in children with ATH who were referred for surgical treatment. Our findings indicate that SDB symptoms are an important determinant of increased overall adiposity and more central fat distribution independent of both NP obstruction severity and BMI. These findings suggest that functional disturbances related to sleep-related breathing may be a more important contributor to abnormal body composition than the severity of anatomical upper airway obstruction.

In our study, 21% of the children were overweight or obese, while 10% were underweight. Similar findings of heterogeneous nutritional status among children with upper airway obstruction caused by ATH have also been reported in other studies [30,31]. Beyond its respiratory manifestations, ATH may adversely affect somatic development. Feeding difficulties, intermittent nocturnal hypoxia, and increased energy expenditure, associated with laboured breathing, have been implicated in the pathogenesis of undernutrition and impaired linear growth [1,32]. However, we did not see linear growth deficits in our cohort. On the other hand, children had significantly higher adiposity than the reference population, as demonstrated by increased skinfold thicknesses, arm and waist circumferences, and mid-upper arm fat area.

ATH and obesity are both known to contribute to paediatric OSAS, but the relative contribution of ATH appears to diminish with increasing age, especially during adolescence [33,34]. Importantly, in analyses adjusted for age and sex, the association between SDB symptoms and increased adiposity remained strong and independent of age. This finding further supports the hypothesis that functional respiratory disorders, rather than anatomical factors, are the main determinants of body composition in children with ATH.

The association of SDB symptoms with increased adiposity may be due to several interconnected mechanisms. Two features of OSAS, fragmented sleep and intermittent hypoxia, can interfere with the neuroendocrine regulation of appetite and energy balance [35,36]. Poor sleep quality and duration are associated with changes in leptin and ghrelin secretion, which increase caloric intake and the preference for energy-dense foods [3,37]. Intermittent hypoxia may also cause metabolic dysregulation by impairing insulin sensitivity and glucose metabolism [38]. Additionally, hypoxia and sleep disruption are associated with low-grade systemic inflammation with increased levels of TNF-α and IL-6 that have a role in obesity and metabolic syndrome [39]. Children with SDB are also frequently affected by daytime fatigue and reduced physical activity, which could result in lower energy expenditure, and behavioural consequences of poor sleep may further impair eating patterns [40]. Collectively, these mechanisms suggest that SDB can be both a consequence and a contributor to obesity, thereby supporting a bidirectional relationship between SDB and altered body composition.

Severe NP obstruction was associated with higher body weight, BMI and selected adiposity measures in univariate analyses but not after adjustment for BMI in the multivariate generalised linear model. Such results indicate that the extent of anatomic disease alone may not fully explain variation in nutritional status, and that sleep-related symptoms may be more reflective of the functional burden of disease. Children with severe NP obstruction had later symptom onset and shorter disease duration than children with moderate NP obstruction, indicating a faster progression of airway compromise in a subset of patients [41]. Our data demonstrate heterogeneity in ATH and emphasise the importance of combining anatomical findings with clinical presentation for a comprehensive assessment. The absence of a strong association between NP obstruction and SDB symptoms supports a multifactorial aetiology of SDB. Craniofacial morphology, neuromuscular tone, body position, and fat distribution are major determinants of airway collapsibility and symptom expression [42,43,44]. Thus, anatomic evaluation alone may underestimate the risk for sleep-related breathing disorders. Consistent with our findings, Seren et al. (2014) did not observe a significant association between adenotonsillar size and OSAS severity but did report associations between ATH, BMI, and snoring sound intensity, suggesting that snoring intensity may serve as a useful adjunct clinical marker [45].

The present study has some limitations. SDB symptoms were assessed by parental report rather than by objective diagnostic procedures such as polysomnography. Symptom assessment was subjective, and it may have lacked the precision of diagnostic testing, leading to potential under- or overestimation of the true prevalence of SDB in this cohort. Second, the cross-sectional design of the study limits us in making any causal conclusions about the relationships between variables. Third, the study was conducted at a single clinical centre and included only children referred for surgical treatment, which may have limited generalisability.

Despite these limitations, the study has several key strengths. Various anthropometric measurements (skinfold thicknesses, circumferences, and indices such as WHtR) were better estimators of nutritional status and body composition than BMI alone. The all-encompassing nature of the approach enabled the assessment and identification of associations with central adiposity, which is closely linked to metabolic risk and adverse long-term health outcomes. Moreover, the agreement of results across different anthropometric indicators further strengthens the robustness of the findings. However, given the observational nature of the study and the subjective assessment of SDB symptoms, these findings should be interpreted with caution. More longitudinal studies with objective SDB assessments and detailed metabolic parameters are needed to determine whether interventions directed at SDB can affect body composition and possibly reduce the risk of metabolic complications in children with ATH.

## 5. Conclusions

Children with ATH and SDB symptoms reported by a parent had increased subcutaneous and central adiposity independent of BMI. In contrast, no significant association was found between adiposity and anatomical severity of nasopharyngeal obstruction. These findings indicate an independent association between SDB symptoms and increased adiposity in children with ATH. However, due to the cross-sectional nature of the study, the directionality and causality of this relationship cannot be established. The results highlight the importance of comprehensive clinical assessment of both SDB symptoms and body composition in children with ATH and underscore the need for longitudinal studies to further elucidate the causal mechanisms underlying these associations.

## Figures and Tables

**Table 1:** Clinical characteristics of patients.

	Boys (n = 57)	Girls (n = 49)	*p* Value
Age (y)	6.04 ± 1.84 (2.5–10.4)	6.12 ± 2.22 (2.5–12.3)	0.848
Age of first symptoms (y)	3.62 ± 2.07 (0.5–9.0)	3.22 ± 2.08 (0.5–9.0)	0.326
Diseases duration:			0.114
≤3y	47 (82.5%)	24 (69.4%)	
>3y	10 (17.5%)	15 (30.6%)	
Adenoid size:			0.086
Small (grades 1–2)	29 (50.9%)	33 (67.4%)	
Large (grade 3)	28 (49.1%)	16 (32.6%)	
Tonsil size:			0.949
Small (grades 0–2)	22 (52.4%)	17 (53.1%)	
Large (3–4)	20 (47.6%)	15(46.9%)	
NP obstruction:			0.282
Moderate (0–3)	22 (38.6%)	24 (49.0%)	
Severe (4–7)	35 (61.4%)	25 (51.0%)	
Previous adenoidectomy or tonsillectomy	8 (14.04%)	6 (12.24%)	0.786
SDB symptoms:			0.954
Yes	16 (28.0%)	14 (28.6%)	
No	41 (72.0%)	35 (71.4%)	
Nutritional status:			0.756
Undernutrition	6 (8.8%)	6 (12.2%)	
Normal weight	39 (68.4%)	34 (69.4%)	
Overweight/Obesity	13 (22.8%)	9 (18.4%)	

NP obstruction—nasopharyngeal obstruction; SDB symptoms—sleep-disordered breathing symptoms.

**Table 2:** Relationships between somatic parameters and disease-related course factors.

Somatic Features (SDS)	Age of First Symptoms (y)	Disease Duration (≤3 y/>3 y)	Adenoid Size (Small/Large)	Tonsil Size (Small/Large)	NP Obstruction Degree (Moderate/Severe)	SDB Symptoms (Yes/No)
Body height	r = 0.04 *p* = 0.719 a	0.203 b	0.899 b	0.073 b	0.187 b	0.497 b
Body weight	r = 0.11 *p* = 0.255 a	0.580 b	0.991 b	0.119 b	0.011 b	0.495 b
BMI	r = 0.12 *p* = 0.207 a	0.989 b	0.897 b	0.311 b	0.008 b	0.192 b
Triceps skinfold thickness	r = 0.15 *p* = 0.119 a	0.162 c	0.717 c	0.931 c	0.039 c	0.298 c
Subscapular skinfold thickness	r = 0.09 *p* = 0.331 a	0.885 c	0.751 c	0.330 c	0.082 c	0.011c
Abdominal skinfold thickness	r = 0.03 *p* = 0.746 a	0.372 c	0.331 c	0.244 c	0.056 c	0.026 c
Sum of 3 skinfold thicknesses	r = 0.14 *p* = 0.165 a	0.304 c	0.479 c	0.357 c	0.023 c	0.037 c
Mid-upper arm circumference (MUAC)	r = 0.07 *p* = 0.499 a	0.568 c	0.953 c	0.150 c	0.095 c	0.149 c
Mid-upper arm area (MUAA)	r = 0.10 *p* = 0.299 a	0.522 b	0.742 b	0.128 b	0.033 b	0.198 b
Mid-upper arm muscle area (MUAMA)	r = 0.03 *p* = 0.720 a	0.425 b	0.439 b	0.233 b	0.687 b	0.906 b
Mid-upper arm fat area (MUAFA)	r = 0.11 *p* = 0.280 a	0.426 c	0.940 c	0.864 c	0.034 c	0.445
Waist circumference	r = −0.007 *p* = 0.944 a	0.209 c	0.280 c	0.078 c	0.035 c	0.416 c
WHtR	r = −0.16 *p* = 0.109 a	0.216 c	0.057 c	0.214 c	0.019 c	0.020 c
Rel. between factors						
Age of first symptoms	-	<0.001 b	0.985 b	0.560 b	<0.001 b	0.491 b
Disease duration		-	0.042 b	0.855 b	<0.001 d	0.292 d
Adenoid size			-	0.684 b	0.005 d	<0.001 d
Tonsil size				-	<0.001 d	0.017 d
NP. obstruction degree					-	0.189 d
SDB symptoms						-

NP obstruction—nasopharyngeal obstruction, SDB symptoms—sleep-disordered breathing symptoms. a—*p* value according to Pearson’s correlation. b—*p* value according to Student’s test. c—*p* value according to the Mann–Whitney test. d—*p* value according to Chi^2^ with Yates’ correction.

**Table 3:** General linear model (GLM) analysis of nutritional status indices and subcutaneous adipose tissue (z-scores) in relation to SDB symptoms and NP obstruction, adjusted for BMI.

Somatic Parameters	Predictors	ß Coefficient	95% CI	*p* Value	Adjusted R^2^
Triceps SFT	SDB symptoms (no/yes)	−0.11	0.01–0.24	0.068	
Triceps SFT	NP obstruction (moderate/severe)	0.04	−0.16–0.08	0.637	0.638
Triceps SFT	BMI (SDS)	0.79	0.67–0.91	0.000	
Subscapular SFT	SDB symptoms (no/yes)	0.25	0.11–0.38	0.000	
Subscapular SFT	NP obstruction (moderate/severe)	0.70	−0.17–0.09	0.553	0.568
Subscapular SFT	BMI (SDS)	1.62	0.57–0.84	0.000	
Abdominal SFT	SDB symptoms (no/yes)	0.19	0.08–0.30	0.000	
Abdominal SFT	NP obstruction (moderate/severe)	−0.06	−0.17–0.06	0.316	0.684
Abdominal SFT	BMI (SDS)	0.80	0.69–0.92	0.000	
Sum of three SFTs	SDB symptoms (no/yes)	0.21	0.11–0.32	0.000	
Sum of three SFTs	NP obstruction (moderate/severe)	−0.06	−0.17–0.05	0.299	0.713
Sum of three SFTs	BMI (SDS)	0.81	0.71–0.92	0.000	
MUAA	SDB symptoms (no/yes)	0.03	−0.09–0.14	0.645	
MUAA	NP obstruction (moderate/severe)	−0.02	−0.14–0.09	0.711	0.669
MUAA	BMI (SDS)	0.83	0.71–0.94	0.000	
MUAFA	SDB symptoms (no/yes)	0.12	0.02–0.23	0.019	
MUAFA	NP obstruction (moderate/severe)	−0.06	−0.17–0.05	0.269	0.720
MUAFA	BMI (SDS)	0.84	0.74–0.95	0.000	
WC	SDB symptoms (no/yes)	0.06	−0.05–0.17	0.256	
WC	NP obstruction (moderate/severe)	−0.05	−0.16–0.07	0.400	0.696
WC	BMI (SDS)	0.84	0.73–0.96	0.000	
WHtR	SDB symptoms (no/yes)	0.21	0.07–0.35	0.003	
WHtR	NP obstruction (moderate/severe)	0.00	−0.14–0.14	0.998	0.528
WHtR	BMI (SDS)	0.69	0.54–0.82	0.000	

NP obstruction—nasopharyngeal obstruction, SDB symptoms—sleep-disordered breathing symptoms, SFT—skinfold thickness, MUAA—mid-upper arm area, MUAFA—mid-upper arm fat area, WC—waist circumference, WHtR—waist-to-height ratio.

## Data Availability

The original contributions presented in this study are included in the article. Further inquiries can be directed to the corresponding author.

## References

[1] Aydogan M., Toprak D., Hatun Ş., Yüksel A., Gokalp A.S. (2007). The Effect of Recurrent Tonsillitis and Adenotonsillectomy on Growth in Childhood. Int. J. Pediatr. Otorhinolaryngol..

[2] Proenca-Modena J.L., Valera F.C.P., Jacob M.G., Buzatto G.P., Saturno T.H., Lopes L., Souza J.M., Paula F.E., Silva M.L., Carenzi L.R. (2012). High Rates of Detection of Respiratory Viruses in Tonsillar Tissues from Children with Chronic Adenotonsillar Disease. PLoS ONE.

[3] Keskin N., Keskin S. (2021). Association between Adenotonsillar Hypertrophy and Leptin, Ghrelin and IGF-1 Levels in Children. Auris Nasus Larynx.

[4] Kaplan B., Kaplan F. (2021). One of the Causes of Adenoid Hypertrophy in Preschool Children, Allergic Rhinitis. Med. Sci. Int. Méd. J..

[5] Yang A., Jv M., Zhang J., Hu Y., Mi J., Hong H. (2023). Analysis of Risk Factors for Otitis Media with Effusion in Children with Adenoid Hypertrophy. Risk Manag. Healthc. Policy.

[6] Xu Z., Wu Y., Tai J., Feng G., Ge W., Zheng L., Zhou Z., Ni X. (2020). Risk Factors of Obstructive Sleep Apnea Syndrome in Children. J. Otolaryngol.-Head Neck Surg..

[7] Jackman A.R., Biggs S.N., Walter L.M., Embuldeniya U.S., Davey M.J., Nixon G.M., Anderson V., Trinder J., Horne R.S.C. (2012). Sleep-Disordered Breathing in Preschool Children Is Associated with Behavioral, but Not Cognitive, Impairments. Sleep Med..

[8] Bhushan B., Ayub B., Loghmanee D.A., Billings K.R. (2015). Metabolic Alterations in Adolescents with Obstructive Sleep Apnea. Int. J. Pediatr. Otorhinolaryngol..

[9] Menzies B., Teng A., Burns M., Lah S. (2022). Neurocognitive Outcomes of Children with Sleep Disordered Breathing: A Systematic Review with Meta-Analysis. Sleep Med. Rev..

[10] Zaffanello M., Ersu R.H., Nosetti L., Beretta G., Agosti M., Piacentini G. (2024). Cardiac Implications of Adenotonsillar Hypertrophy and Obstructive Sleep Apnea in Pediatric Patients: A Comprehensive Systematic Review. Children.

[11] Marcus C.L., Brooks L.J., Draper K.A., Gozal D., Halbower A.C., Jones J., Schechter M.S., Ward S.D., Sheldon S.H., Shiffman R.N. (2012). Diagnosis and Management of Childhood Obstructive Sleep Apnea Syndrome. Pediatrics.

[12] Li A.M., Wong E., Kew J., Hui S., Fok T.F. (2002). Use of Tonsil Size in the Evaluation of Obstructive Sleep Apnoea. Arch. Dis. Child..

[13] Hwang S.-H., Guilleminault C., Park C.-S., Kim T.-W., Hong S.-C. (2013). Usefulness of Adenotonsillar Size for Prediction of Severity of Obstructive Sleep Apnea and Flow Limitation. Otolaryngol. Head Neck Surg..

[14] Dayyat E., Kheirandish-Gozal L., Capdevila O.S., Maarafeya M.M.A., Gozal D. (2009). Obstructive Sleep Apnea in Children Relative Contributions of Body Mass Index and Adenotonsillar Hypertrophy. Chest.

[15] Tagaya M., Nakata S., Yasuma F., Miyazaki S., Sasaki F., Morinaga M., Suzuki K., Otake H., Nakashima T. (2012). Relationship between Adenoid Size and Severity of Obstructive Sleep Apnea in Preschool Children. Int. J. Pediatr. Otorhinolaryngol..

[16] Danisi J.M., Fernandez-Mendoza J., Vgontzas A.N., Calhoun S.L., He F., Liao D., Bixler E.O. (2020). Association of Visceral Adiposity and Systemic Inflammation with Sleep Disordered Breathing in Normal Weight, Never Obese Adolescents. Sleep Med..

[17] Burton Z.A., Nissenbaum C., Singh M., Ball C., Khokar G., Bhargava E.K., Elphick H.E. (2025). An Exploratory Retrospective Study of Clinical Outcomes Following Adenotonsillectomy in Children with Obstructive Sleep Disordered Breathing Living with Severe Obesity. Clin. Otolaryngol..

[18] Bhatia R., Lesser D.J., Oliveira F.G.S.A., Tran W.H., Keens T.G., Khoo M.C.K., Ward S.L.D. (2015). Body Fat Composition: A Predictive Factor for Sleep Related Breathing Disorder in Obese Children. J. Clin. Sleep Med..

[19] Lopes L.L.d.A., Costa F.W.G., Cevidanes L.H.S., Silva P.G.d.B., Gurgel M.L., Carvalho F.S.R., Júnior C.M.C., Ribeiro T.R. (2024). Anthropometric Measures and Obstructive Sleep Apnea in Children and Adolescents: A Systematic Review of the Literature and Meta-Analysis. Sleep Breath..

[20] Toros S.Z., Noşeri H., Ertugay Ç.K., Külekçi S., Habeşoğlu T.E., Kılıçoğlu G., Yılmaz G., Egeli E. (2010). Adenotonsillar Hypertrophy: Does It Correlate with Obstructive Symptoms in Children?. Int. J. Pediatr. Otorhinolaryngol..

[21] Wang J., Zhao Y., Yang W., Shen T., Xue P., Yan X., Chen D., Qiao Y., Chen M., Ren R. (2019). Correlations between Obstructive Sleep Apnea and Adenotonsillar Hypertrophy in Children of Different Weight Status. Sci. Rep..

[22] Yetim M., Kalçık M., Bekar L., Karavelioğlu Y., Yılmaz Y.A. (2025). Body Composition Analysis in Obstructive Sleep Apnea: A Cross-Sectional Study Using Bioelectrical Impedance Analysis. Clin. Respir. J..

[23] Mitchell R.B., Archer S.M., Ishman S.L., Rosenfeld R.M., Coles S., Finestone S.A., Friedman N.R., Giordano T., Hildrew D.M., Kim T.W. (2019). Clinical Practice Guideline: Tonsillectomy in Children (Update). Otolaryngol. Head Neck Surg..

[24] Sharifkashani S., Dabirmoghaddam P., Kheirkhah M., Hosseinzadehnik R. (2015). A New Clinical Scoring System for Adenoid Hypertrophy in Children. Iran. J. Otorhinolaryngol..

[25] Brodsky L. (1989). Modern Assessment of Tonsils and Adenoids. Pediatr. Clin. N. Am..

[26] Palczewska I., Niedźwiecka Z. (2001). Wskaźniki Rozwoju Somatycznego Dzieci i Młodzieży Warszawskiej. Dev. Period Med..

[27] Świąder-Leśniak A., Kułaga Z., Grajda A., Gurzkowska B., Góźdź M., Wojtyło M., Różdżyńska-Świątkowska A., Mieczysław L. (2015). Wartości Referencyjne Obwodu Talii i Bioder Polskich Dzieci i Młodzieży w Wieku 3-18 Lat [References for Waist and Hip Circumferences in Polish Children and Adolescents 3-18 Year of Age]. Stand. Med./Pediatr..

[28] Cole T.J., Bellizzi M.C., Flegal K.M., Dietz W.H. (2000). Establishing a Standard Definition for Child Overweight and Obesity Worldwide: International Survey. BMJ.

[29] Cole T.J., Flegal K.M., Nicholls D., Jackson A.A. (2007). Body Mass Index Cut Offs to Define Thinness in Children and Adolescents: International Survey. BMJ.

[30] Dualibi A.P.F.F., Pignatari S.S.N., Weckx L.L.M. (2002). Nutritional Evaluation in Surgical Treatment of Children with Hypertrophic Tonsils and or Adenoids. Int. J. Pediatr. Otorhinolaryngol..

[31] Daar G., Sarı K., Gencer Z.K., Ede H., Aydın R., Saydam L. (2016). The Relation between Childhood Obesity and Adenotonsillar Hypertrophy. Eur. Arch. Oto-Rhino-Laryngol..

[32] Esteller E., Villatoro J.C., Agüero A., Lopez R., Matiñó E., Argemi J., Girabent-Farrés M. (2018). Obstructive Sleep Apnea Syndrome and Growth Failure. Int. J. Pediatr. Otorhinolaryngol..

[33] Kang K.-T., Chou C.-H., Weng W.-C., Lee P.-L., Hsu W.-C. (2013). Associations between Adenotonsillar Hypertrophy, Age, and Obesity in Children with Obstructive Sleep Apnea. PLoS ONE.

[34] Kaditis A.G., Alexopoulos E.I., Hatzi F., Karadonta I., Chaidas K., Gourgoulianis K., Zintzaras E., Syrogiannopoulos G.A. (2008). Adiposity in Relation to Age as Predictor of Severity of Sleep Apnea in Children with Snoring. Sleep Breath..

[35] Canapari C.A., Hoppin A.G., Kinane T.B., Thomas B.J., Torriani M., Katz E.S. (2011). Relationship between Sleep Apnea, Fat Distribution, and Insulin Resistance in Obese Children. J. Clin. Sleep Med..

[36] Roche J., Corgosinho F.C., Isacco L., Scheuermaier K., Pereira B., Gillet V., Moreira G.A., Pradella-Hallinan M., Tufik S., Mello M.T.d. (2020). A Multidisciplinary Weight Loss Intervention in Obese Adolescents with and without Sleep-Disordered Breathing Improves Cardiometabolic Health, Whether SDB Was Normalized or Not. Sleep Med..

[37] van Egmond L.T., Meth E.M.S., Engström J., Ilemosoglou M., Keller J.A., Vogel H., Benedict C. (2023). Effects of Acute Sleep Loss on Leptin, Ghrelin, and Adiponectin in Adults with Healthy Weight and Obesity: A Laboratory Study. Obesity.

[38] Narang I., McCrindle B.W., Manlhiot C., Lu Z., Al-Saleh S., Birken C.S., Hamilton J. (2018). Intermittent Nocturnal Hypoxia and Metabolic Risk in Obese Adolescents with Obstructive Sleep Apnea. Sleep Breath..

[39] Bhatt S.P., Guleria R., Kabra S.K. (2021). Metabolic Alterations and Systemic Inflammation in Overweight/Obese Children with Obstructive Sleep Apnea. PLoS ONE.

[40] Spruyt K., Capdevila O.S., Serpero L.D., Kheirandish-Gozal L., Gozal D. (2010). Dietary and Physical Activity Patterns in Children with Obstructive Sleep Apnea. J. Pediatr..

[41] Sakoda-Iwata R., Iwasaki T., Tsujii T., Hisagai S., Oku Y., Ban Y., Sato H., Ishii H., Kanomi R., Yamasaki Y. (2023). Does Rapid Maxillary Expansion Improve Nasal Airway Obstruction? A Computer Fluid Dynamics Study in Patients with Nasal Mucosa Hypertrophy and Obstructive Adenoids. Am. J. Orthod. Dentofac. Orthop..

[42] Valera F.C.P., Travitzki L.V.V., Mattar S.E.M., Matsumoto M.A.N., Elias A.M., Anselmo-Lima W.T. (2003). Muscular, Functional and Orthodontic Changes in Pre School Children with Enlarged Adenoids and Tonsils. Int. J. Pediatr. Otorhinolaryngol..

[43] Marcus C.L., Katz E.S., Lutz J., Black C.A., Galster P., Carson K.A. (2005). Upper Airway Dynamic Responses in Children with the Obstructive Sleep Apnea Syndrome. Pediatr. Res..

[44] Ikävalko T., Närhi M., Eloranta A.-M., Lintu N., Myllykangas R., Vierola A., Tuomilehto H., Lakka T., Pahkala R. (2017). Predictors of Sleep Disordered Breathing in Children: The PANIC Study. Eur. J. Orthod..

[45] Seren E., San T., Cingi C., Muluk N.B., Durukan K. (2014). Effects of Body Mass Index and Adenotonsillar Size on Snoring Sound Intensity Levels at Highest Power. Int. J. Pediatr. Otorhinolaryngol..

